# Extra-Mediterranean refugia: The rule and not the exception?

**DOI:** 10.1186/1742-9994-9-22

**Published:** 2012-09-06

**Authors:** Thomas Schmitt, Zoltán Varga

**Affiliations:** 1 Biogeography, Trier University, D – 54 286, Trier, Germany; 2 Dept. Evolutionary Zoology, Faculty of Science and Technology, University of Debrecen, Egyetem-tér 1, Debrecen, H-4010, Hungary

**Keywords:** Phylogeography, Refugia, Faunal types, Last Glacial Maximum (LGM), Postglacial, Range expansions, Range shifts, Mediterranean, Continental, Siberian

## Abstract

Some decades ago, biogeographers distinguished three major faunal types of high importance for Europe: (i) Mediterranean elements with exclusive glacial survival in the Mediterranean refugia, (ii) Siberian elements with glacial refugia in the eastern Palearctic and only postglacial expansion to Europe and (iii) arctic and/or alpine elements with large zonal distributions in the periglacial areas and postglacial retreat to the North and/or into the high mountain systems. Genetic analyses have unravelled numerous additional refugia both of continental and Mediterranean species, thus strongly modifying the biogeographical view of Europe. This modified notion is particularly true for the so-called Siberian species, which in many cases have not immigrated into Europe during the postglacial period, but most likely have survived the last, or even several glacial phases, in extra-Mediterranean refugia in some climatically favourable but geographically limited areas of southern Central and Eastern Europe. Recently, genetic analyses revealed that typical Mediterranean species have also survived the Last Glacial Maximum in cryptic northern refugia (e.g. in the Carpathians or even north of the Alps) in addition to their Mediterranean refuge areas.

## Introduction

The biogeography of the western Palearctic is quite complex and therefore a fascinating and challenging research subject [1-8]. Scientists, even about 50 years ago, distinguished three major faunal components in Europe (Mediterranean, Siberian, arctic and/or alpine), but the interpretation of the underlying biogeographical processes behind these faunal elements has considerably changed since then, e.g. [8-13]. Furthermore, the understanding of climatic and other environmental conditions during glaciations has substantially deepened, e.g. [14-19].

**Figure 1:**
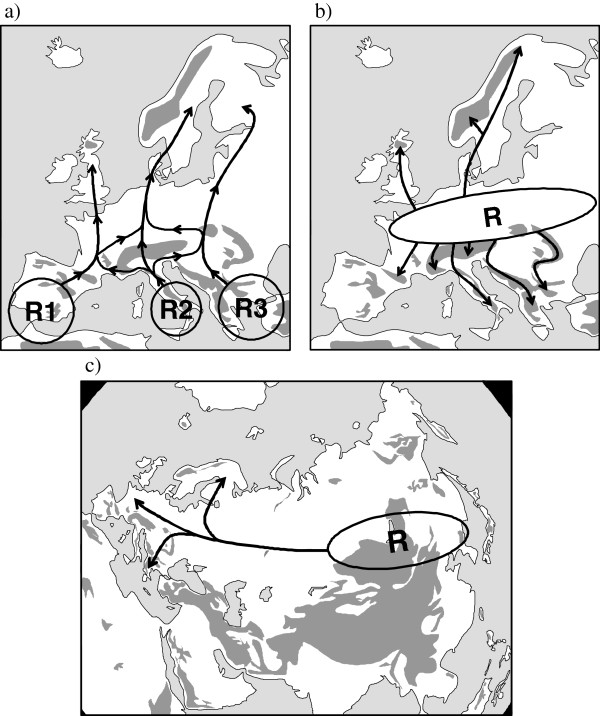
** The interpretation of glacial refugia (indicated by "R") and postglacial range changes (indicated by arrows) of the three most important biogeographical elements of Europe (a: Mediterranean, b: arctic/alpine, c: Siberian/Manchurian) as suggested by de Lattin.**[4]**, simplified**

By the time of de Lattin [4], the existence of Mediterranean faunal elements was largely acknowledged (e.g. the “holothermic” faunal elements of Rebel [20]). These elements were thought to have exclusively survived the ice ages in the Mediterranean region, which was divided into nine sub-centres [21] composed of several core areas (German: Arealkerne [2]). Depending on the postglacial expansion out of these refugia and differentiation centres, two basic types were distinguished: (i) stationary elements which did not essentially enlarge their distributions northwards during the postglacial period and (ii) expansive elements largely expanding their ranges beyond their Mediterranean refuge areas, frequently as far north as southern Scandinavia and often showing peripherally isolated populations (subspecies) at the northern boundary of their range e.g. [4] (Figure 1a).

Furthermore, the existence of large ice age distributions in the zonal periglacial belt was suggested for the species with arctic, alpine or arctic-alpine distributions followed by postglacial retreat to high mountain areas in the South and/or the high latitudes in the North. Retreat into both directions was interpreted as the reason for the arctic-alpine disjunctions today [22,23]. However, local endemics e.g. in the Alps were interpreted (at least partly) as *in situ* survival e.g. at nunataks [23] and/or in some marginal areas of the Alps (“massifs de refuge”, [24]) and other southern European high mountain systems [25,26]. Thus, the more widespread species in this group were interpreted, following the old monoglacial ideas of Scharff [27], as the only species surviving north of the mountain chains of the Pyrenees, Alps and Carpathians (Figure 1b).

**Figure 2:**
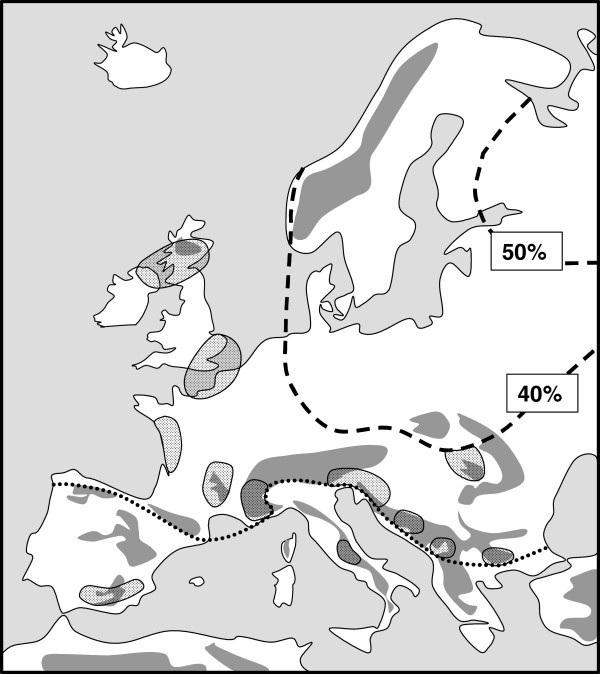
** European accumulation zones of marginal subspecies of species showing the continental distribution type (shaded areas).** Most of these areas were later discovered as being important as extra-Mediterranean centres of glacial survival of thermophilic species. The southern limits of some of these zones coincide with the line of packing of the "Siberian" faunal type in Europe (sensu de Lattin [4]; dotted line). The representation of "Siberian" species in the local faunal lists is given as broken lines with indication of their respective percentages. Redrawn after de Lattin [4] and Varga [39].

The third biogeographical unit, the large group of species with continental distributions (i.e. missing or rather fragmented in the Mediterranean parts of Europe and absent in the vicinity of the Atlantic) was interpreted as completely missing in Europe during the ice ages, but surviving in the eastern Palearctic in Siberian and/or Manchurian refugia [28]. A postglacial and thus very rapid colonisation of these species throughout Asia to Europe was postulated (Figure 1c) with a typical “line of packing” (i.e. the accumulation of distribution borders; German: Stauungslinie) at the margin of the Mediterranean area (Figure 2). However, this point of view was strongly questioned since the 1960-70ies because this “line of packing” was recognised as an accumulation area of peripheral subspecies of continental species and was also re-considered and interpreted as possible survival areas of such species in some areas of south-central and south-eastern Europe (Figure 2; [25,29-33]). Since then, evidence has accumulated for various extra-Mediterranean refugia in Europe, e.g. [8,9,11-13,34-38].

Over the past two decades genetic analyses of many animal and plant species, representing different biogeographical groups, have strongly enhanced our understanding of the highly complex biogeographical patterns and processes within the western Palearctic [5-8,10,40-44]. Therefore, we review the changing view with respect to extra-Mediterranean refugia with a special focus on the geographic location of these refugia, their mostly limited spatial extent and often cryptic natures. As recent reviews already address these extra-Mediterranean refugia in artic/alpine species [10,45-48], we focus in this article on continental and Mediterranean species.

## Continental species: less mobile but much more complex and diverse

The species with typical continental distribution patterns (i.e. not reaching the areas with typical Atlantic and Mediterranean climates, but being widely distributed from continental Europe throughout temperate Asia) were formerly thought to have immigrated to Europe from eastern Asiatic Würm glacial refuge areas (i.e. from the Siberian and the Manchurian core areas) during the postglacial period (Figure 1c) [28]. However, this idea was questioned afterwards on the basis of chorological (i.e. the classical interpretation of distribution patterns) and intraspecific taxonomical analyses (cf. Figure 2). Consequently, survival of such continental species was postulated in Europe outside the classical Mediterranean refuge areas [25,29-33].

**Figure 3:**
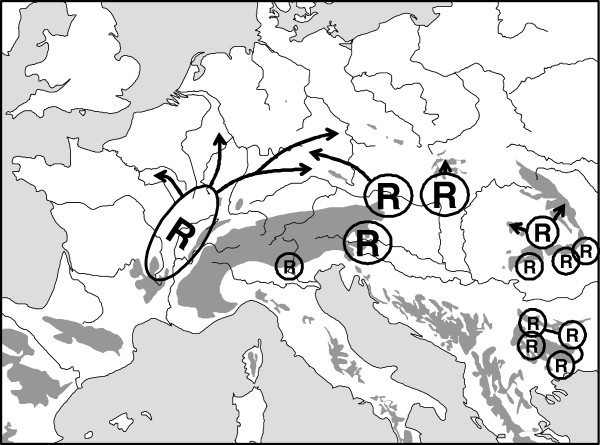
** Possible Würm glacial distribution patterns (indicated by "R") of the butterfly*Erebia medusa*and assumed postglacial range changes (arrows).** Overlapping refuge symbols or lines between them indicate possible gene flow between them during the last ice age or even a larger structured refuge area (especially in the case of Bulgaria). Redrawn after Schmitt & Seitz [51], Schmitt *et al*. [50] and Hammouti *et al*. [53].

One of the best studied examples of these continental species is the woodland ringlet *Erebia medusa*[49-53]. Allozyme analyses over major parts of the species’ European distribution range showed strong differentiation into a variety of different genetic lineages: three in Central and East Europe, one in the southern Alps, four or more in and around the Carpathian Basin and one with remarkable sub-structures in Bulgaria [50,51]. Analyses of the mtDNA locus COI of the samples from Central and East Europe, the southern Alps and the western Carpathian Basin strongly supported the genetic lineages detected with allozyme electrophoreses [53]. These results confirm the idea of a larger number of extra-Mediterranean Würm ice age refuge areas in Europe and not postglacial immigration to Europe out of Asiatic core areas. These data strongly support such refugia having been localised (i) west of the Alps, (ii) south of the Alps, (iii) east/south-east of the Alps, (iv) south of the northern Carpathians, (v) in the eastern Carpathian Basin between the Apuseni Mts. and the eastern Carpathians, (vi) on the southern slopes of the southern Carpathians and (vii) in the vicinity of the Bulgarian mountain systems, the latter most probably in a structured distribution possibly with temporal disjunctions during some periods of the last ice age (Figure 3). Most of these refugia are assumed to be geographically small and situated in climatically buffered pockets in the landscape cf. [9]. Although numerous morphological subspecies of *E. medusa* have been described, in some cases even supporting these genetic patterns, the refugia postulated on allozyme patterns are even more numerous and are geographically more informative and concise than the known phenotypically differentiated groups. Also, they cannot be deduced from the actual, more or less continuous, distribution pattern of *E. medusa*. Therefore, the only adequate possibility to comprehensively asses the complex biogeography and refugial structure of this species is through an analysis of its genetic patterns.

**Figure 4:**
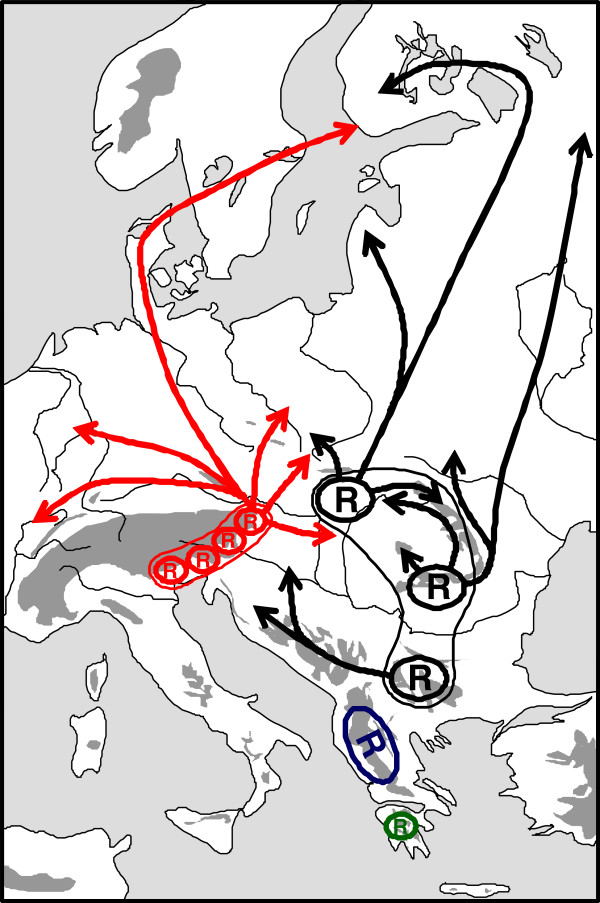
** Possible Würm glacial distribution patterns of the butterfly*Parnassius mnemosyne*, eastern lineage (refuge areas with closely related haplotypes are grouped and in the same colour) and assumed postglacial range changes (arrows) in eastern and south-eastern Europe.** Redrawn after Gratton *et al*. [54].

A similar situation was observed in the eastern phylogenetic clade of the clouded apollo *Parnassius mnemosyne* by an analysis of mtDNA sequences of the COI locus over large parts of eastern and south-eastern Europe [54]. This analysis also supported a larger number of extra-Mediterranean Würm ice age refugia from the lower parts of the northern Carpathians and the eastern Alps forelands throughout the Carpathian Basin to the extra-Mediterranean regions of the Balkan Peninsula (Figure 4). The Carpathian Basin shows the overlap of three main haplotype groups of the eastern lineage of this polytypic species. At least one of them has expanded northwards from the Carpathian region reaching southern Finland.

A complex hierarchical pattern was also observed in the adder *Vipera berus*, a snake distributed from western Europe throughout Asia as far east as the island of Sakhalin. Ursenbacher *et al*. [55] showed a strong differentiation into three major genetic lineages of most probably pre-Pleistocene origin with two of these being geographically restricted (northern Italian Alps; western Balkan mountains) and one being fairly widespread from France and the UK to the Pacific. The widespread lineage is further divided into several sub-clades of which all apart from one are confined to some part of Europe. These data suggest that two relict lineages most probably survived long periods of time (maybe even the whole Pleistocene) in extra-Mediterranean regions at the southern Alps margin and in the mountainous regions of the western Balkan Peninsula.

**Figure 5:**
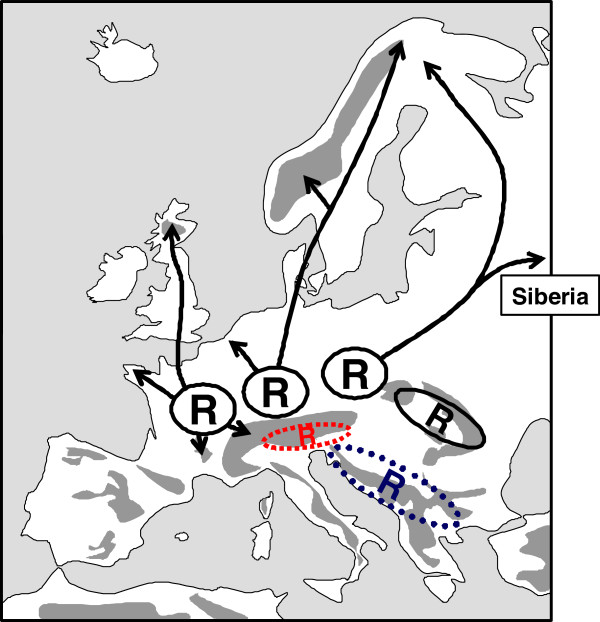
** Possible Würm glacial distribution patterns (indicated by "R") of the adder*Vipera berus*and assumed postglacial range changes (arrows).** The three major genetic lineages are indicated by different signatures of the refugia – dotted line, blue: western Balkan lineage; broken line, red: south-eastern Alps line; solid line, black: widespread lineage with different sub-lineages. Redrawn after Ursenbacher *et al*. [55].

The sub-clades in the widespread lineage persisted during at least the last ice age (and maybe even evolved) in other extra-Mediterranean refugia in France, north of the Alps and in the Carpathian Basin. Northern Europe and Asia were postglacially colonised from the northern and north-eastern most lineages, respectively. The refugial populations in France contracted from their ice age distribution to the northern parts of the country and to the British Isles, as well as to the higher elevations (e.g. Massif Central) in southern France (Figure 5).

The sibling *Vipera seoanei*, restricted to the mountain ranges of northern Iberia, is even more strongly differentiated from *V. berus* than the lineages within this latter species [55]. The split between these two species probably occurred before the beginning of the glacial-interglacial cycles, with *V. seoanei* most likely having continuously existed over time in the extra-Mediterranean regions of Iberia. It is in this area that the species has two morphologically differentiated subspecies [56], thus indicating two centres of glacial survival.

Furthermore, mtDNA analyses of the snail *Arion fuscus* support an extra-Mediterranean survival of this species especially in the eastern regions of the Alps, but maybe also in other extra-Mediterranean retreats such as in the area of the Tatras [57]. A strongly differentiated lineage of this species is restricted to the Balkan Peninsula. Whether the respective refugia have to be considered Ponto-Mediterranean or Balkan extra-Mediterranean is in need of further genetic analyses.

**Figure 6:**
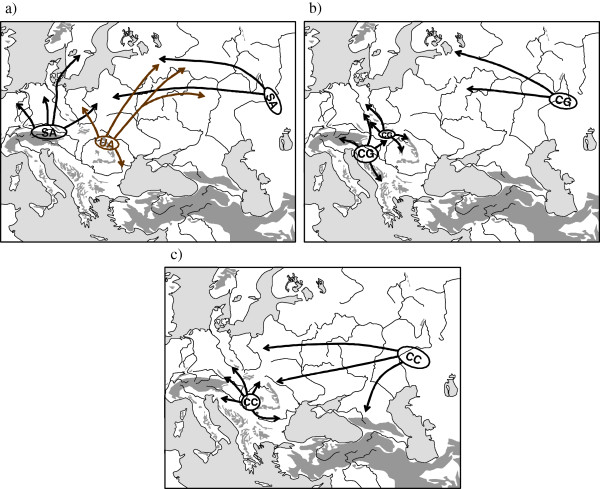
** Extra-Mediterranean refugia in eastern Europe and their postglacial expansions of (a)*Sorex araneus*(SA, black),*Ursus arctos*(UA, brown), (b)*Clethrionomys glareolus*(CG) and (c)*Cricetus cricetus*(CC).** Redrawn after Polyakov *et al*. [67], Hofreiter *et al*. [68], Hedrick & Waits [69], Neumann *et al*. [70], Saarma *et al*. [62], Valdiosera *et al*. [71], Ho *et al*. [72], Krause *et al*. [73], Korsten *et al*. [74] and Ledevin *et al*. [75].

The particular importance of a Carpathian refugium as extra-Mediterranean retreat has repeatedly been suggested in several groups of vertebrates, e.g. in the moor frog (*Rana arvalis*), the agile lizard (*Lacerta agilis*), the bank vole (*Clethrionomys glareolus*), the common and the field vole (*Microtus arvalis, M. agrestis*), the wild boar (*Sus scrofa*), the roe deer (*Capreolus capreolus*) and the red deer (*Cervus elaphus*), and even in the brown bear (*Ursus arctos*), e.g. [34,35,58-64] (Figure 6). In the lizard *Zootoca vivipara*, a special mtDNA haplotype restricted to northern Hungary and Austria was found. This ovo-viviparous form shows peculiar karyotypic characters and demonstrates that the ovo-vivipary has independently evolved in the western European and Pannonian (*Zootoca vivipara pannonica*) populations [65,66].

Surveys of the bank vole (*Clethrionomys glareolus*) have not only shown the significance of “northern” refugia in the Carpathians, but also in some other areas, e.g., the vicinity of the Alps, southern France, and southern parts of the Ural Mountains (Figure 6b). New fossil and genetic records support the refugial character of the southern Urals in eastern Europe. Recently, a morphometric analysis of bank vole molars has unravelled the existence of a “Ural” morphotype of this species [75].

Although the transitional forest steppic character of the LGM vegetation of the Russian plain already was demonstrated in several palaeo-ecological publications e.g. [76,77], the importance of the refugia in the Urals (Figure 6), also in Kazakhstan and south-western Siberia was only recently shown [67,78,79], and Danukalova *et al*. [78] pointed out that “*the changes of the palaeoenvironment were not so sharp as in the adjacent northwestern territories. Biota of the region has been formed under the influence of the European and Asiatic elements*”.

For common shrews (*Sorex araneus*), two major continental refugia have been discovered, one in the southern Urals, from which the re-population of northern Europe started, and a southern Siberian core area which shows a geographic co-incidence with some nemoral species ([67]; further details e.g. in Walter and Straka [80]; Walter and Breckle [81]). These results have been repeatedly confirmed by the karyological and molecular analyses of Wóycik *et al*. [82], and also perfectly harmonise with the suggested history of postglacial expansion of *C. glareolus* from a southern continental core area (Urals) to northern Russia [75]. Additionally, in forest bird species, the Caucasus appears to be an area of genetic divergence. Endemic (or nearly so) clusters of haplotypes in the Caucasus have been documented for several species (*Carpodacus erythrinus, Motacilla albaSitta europaeaTroglodytes troglodytes*; reviewed by Zink *et al*. [83]).

All these examples clearly show that the continental species have had much more refugia and thus, performed much smaller range shifts and expansions than previously thought. Expansion from Siberian core areas into Europe have only been demonstrated in mobile species of boreo-temperate forests such as the great spotted woodpecker (*Dendrocopus maior*), the boreal warbler (*Phylloscopus borealis*), the Siberian flying squirrel (*Pteromys volans*), or the wood lemming (*Myopus schisticolor*) [84-87]. These species exhibit a higher level of genetic differentiation only at the eastern-southeastern parts of their distributions. Additionally, the dwarf damselfly *Nehalennia speciosa*, a specialist species of oligotrophic peat bogs, shows a very shallow differentiation all over the Palearctic and most probably expanded from an eastern Asiatic ice age refuge at the beginning of the postglacial period [88]. Some species of boreal forests have had at least two subsequent waves of expansion from the eastern Palaearctic to Europe, from which the representatives of the earlier ones have been preserved in some southern European high mountains as the Pyrenees, the Cantabrian mountains and/or in the Balkans, as e.g. the capercaille [89,90].

For Holarctic boreal forest species, a deeper split has only been shown between the Nearctic and the Palearctic populations (e.g. the birds *Picoides tridactylus, Pinicola enucleator, Troglodytes troglodytes*), mostly connected with stronger genetic differentiation in the Nearctic [84,91,92]. Therefore, a direct Siberian invasion to Europe is rare and can only be supported for some boreal forest species by shallow phylogeographic structures. This pattern might be the strict exception in temperate non-forest species and not one of the paradigms as postulated by de Lattin [28].

In general, these extra-Mediterranean refugia were apparently often located in the vicinity of water donating mountains systems as the glaciated Alps, Carpathians or Balkan mountain systems cf. [51], which may have received more precipitation during the kryoxerotic LGM than the adjacent lowland loess steppe areas. The same idea implicitly appears in some recent papers of Bhagwat and Willis [36], Varga [13] and Stewart *et al.*[12] supported by habitat preference data of numerous, mostly woody plant and vertebrate species. Furthermore, many of these refugia must have been small and sporadic in their geographic extent [93,94] so that they have been overlooked in the past reconstruction of the glacial faunas mostly based on fossil records. This might explain their cryptic character, which is in clear contrast to their great importance for the re-colonisation of major parts of Europe, a fact that could only be demonstrated by the recent genetic analyses.

## Simultaneous ice age survival in Mediterranean and extra-Mediterranean refugia of temperate species

De Lattin’s paradigm [21] of survival of thermophilic animal and plant species exclusively in Mediterranean Würm refugia remained untouched until quite recently. However, evidence has accumulated that glacial distribution patterns were considerably more intricate than previously thought [8,38], and even additional extra-Mediterranean ice age refugia are looking more and more likely for some of these Mediterranean taxa [11,12]. The combination of southern and continental refugia became especially evident in the often cited case of the “paradigmatic” brown bear, e.g. [42,62,74,95]. The successful extraction and sequencing of mtDNA from fossil bones has shown the highly complex phylogeographic pattern of this species [68,69,71-73]. Thus, the predictions of the “expansion-contraction” (E/C) model were not supported, and consequently the classic glacial refugium model is insufficient to explain the genetic history of European brown bears.

**Figure 7:**
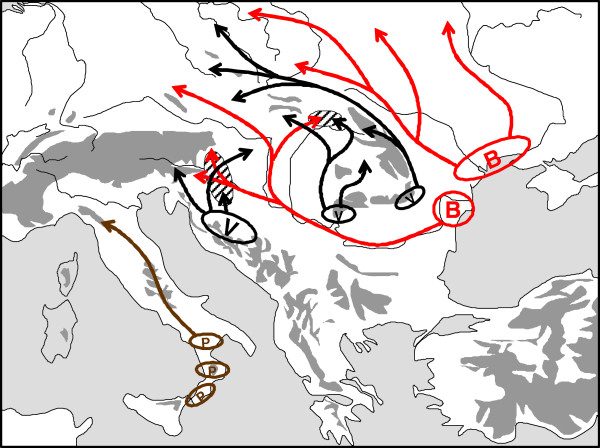
** Possible Würm glacial distribution patterns of fire-bellied toads (indicated by "B" (in red) for*Bombina bombina*, "V" (in black) for*B. variegata*and "P" (in brown) for*B. pachypus*) and assumed postglacial range changes (arrows) in Europe.** Areas of sympatry of both species are indicated by hatched areas. Redrawn after Szymura *et al*. [96], Canestrelli *et al*. [97], Vörös *et al*. [98] and Hofman *et al*. [99].

The Pleistocene glacial refugia of the European *Bombina* toads were located both in the “classical” refugial areas of the Apennines and the Balkans (core areas of *B. pachypus* and subspecies of *B. variegata*; [96,97]) as well as more to the North, in the Carpathians and the adjoining lowlands [98,99]. Also, strong genetic evidence shows that *B. variegata* survived the LGM in climatically favourable regions in the southern Carpathians. The mtDNA and allozyme data suggest two separate refugia. One clade probably had its refugium in the south-eastern edge of the Carpathians, while the most likely refugium of the other clade was in the area of the southern Carpathians where the highest haplotype diversity was detected (Figure 7). However, the deep genetic overall divergence among European *Bombina* lineages suggests their pre-Pleistocene origin. A similar south–north duplicity was found in the common hamster (*Cricetus cricetus*) in which a Pannonian and a northern clade have been distinguished [70] with tracks of expansion (i) crossing the “Porta Hungarica” into southern Czech Republic (Moravia), and (ii) north of the Carpathian arc into northern Central Europe (Figure 6c).

Furthermore, Fink *et al*. [100] found a genetic lineage in the rodent *Microtus arvalis*, endemic to the region of the Black Forest (south-western Germany). Tougard et al. [63] described two more lineages of this species, which most likely have evolved in extra-Mediterranean refugia of Central and East Europe, including the Carpathian Basin. Consequently, *M. arvalis* survived at least the last ice age in a complex system of extra-Mediterranean and classical Mediterranean core areas. However, we have to assume that the more northern part of the glacial distribution of this vole was, at least transitionally, restricted to geographically small regions with a more buffered climate, thus only some extra-zonal areas in the periglacial tundra and steppe region, although *M. arvalis* in general was a common component of fossil remains of last glacial faunas [101].

**Figure 8:**
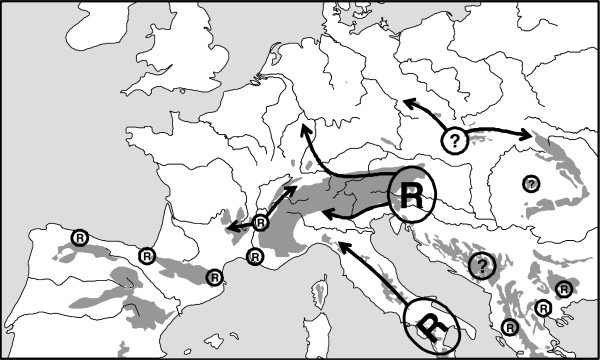
** Possible Würm glacial distribution patterns (indicated by "R"; "?" is used in cases of still doubtful refuge areas) of the beech,*Fagus sylvatica*, and assumed postglacial range changes (arrows).** Redrawn after Magri *et al*. [102] and Magri [103].

The highly important recent work on the phylogeography of the beech, *Fagus sylvatica*, [102,103] strongly suggest a larger number of additional but geographically restricted extra-Mediterranean Würm glacial refugia for this species, which beforehand had always been considered to be of pure Mediterranean origin cf. [6]. Thus, retreats were demonstrated in Mediterranean southern Italy and in a number of geographically restricted areas in the Balkan Peninsula, of which some even should have been located in the continental extra-Mediterranean part of this peninsula. A similar pattern is true for Iberia where the retreats were most probably located along the northern mountain chains of the Cordillera Cantabrica and thus in the extra-Mediterranean area of this region. Further extra-Mediterranean refugia of the beech are likely in two areas of southern France (with one in the vicinity of the Massif Central), the eastern Alps and, maybe, the Carpathian Basin (Apuseni Mts.) and the northern Carpathians (Figure 8). Furthermore, refugia for some other but more cold-tolerant tree species were frequently proved for the Carpathian region and the Carpathian Basin by classical pollen analyses and macrofossil surveys, e.g. [37,38,104-112].

## General discussion and conclusions

A large number of recent publications have shown the great importance of extra-Mediterranean refugia for temperate species and not only for alpine and arctic taxa. While the latter two groups may have frequently had wide ranges over this cold-continental zonobiome [10], the glacial range contractions of thermophilic temperate species, in most cases, must have led to small (and very small) meso- or microclimatically favourable extra- or intrazonal areas within the extended periglacial belt.

Many species with typical continental distributions might have had glacial distribution patterns with multiple extra-Mediterranean refugia, and were mostly not restricted to refuge areas in the eastern Palearctic, as often previously thought. However, this is not to say that they were absent from the eastern Palearctic, but these contractions were not the exclusive ones for these species.

Instead, recent research showed that even thermophilic species, which were formerly thought of having been completely restricted to Mediterranean core areas, could in some cases survive in extra-Mediterranean refugia in addition to the typical Mediterranean areas. Such populations in many cases have an even higher genetic diversity and expansive power than populations restricted to the more southern “classical” refugia.

These observations can be explained by two different factors, which might have acted in combination. The southern refugia of temperate species were often surrounded by extended cold-arid steppe areas, e.g. in the central part of the Balkan Peninsula and also in the Carpathian Basin [113]. Furthermore, the populations of the scattered extra-Mediterranean refugial pockets could expand and hybridise among each other during the milder interstadial phases of the Würm and also between the LGM and the younger Dryas period [12]. It means that these extra-Mediterranean refuge populations have survived at the rear edge of the range during the ice ages, with all evolutionary consequences of this situation [114]. These ice age rear edges became the leading edges of the postglacial northwards range expansions, thus strongly impacting the genetic constitution of Central and North Europe in many plant and animal species. In many cases, such populations have been characterised as localised subspecies of extended polytypic continental species, and they are considered as evolutionarily significant units (ESUs) of high conservation priority [95].

For all these reasons, the extra-Mediterranean refugia apparently represent an important biogeographical component of the western Palaearctic, maybe nearly equivalent to the Mediterranean refugia further south.

Through these new findings, we can also answer the question of the article’s title: whether extra-Mediterranean refugia are the rule or the exception. In fact, they are a bit of both. Extra-Mediterranean refugia have been a common feature during, at least, the last ice age and thus are paradigmatic. However, they are also represented by many individual patterns of particular biogeographical features so that each case shows at least some uniqueness. The principle of individual responses of the species to climatic oscillations between glacial and interglacial conditions was repeatedly postulated in this context (cf. Stewart *et al*., [12], but also see Bhagwat & Willis [36]). During glaciations, ecosystems that exist today had been largely disintegrated and were represented by *de novo* ecosystems without close connection with the succeeding ones (e.g. the mammoth steppe with tundra, cold steppic and alpine elements). These ecosystems were locally intermingled with small forest refugia (i.e. the pockets of forests of Bhagwat & Willis, [36]) and also showed non-analogous mammal assemblages [13,35,77,115].

Therefore, no regular North–South shifts took place between glacial and interglacial conditions and *vice versa* (as implicitly assumed in the biogeographical range paradigms, cf. [5,6]). Instead, a new sequence of ecosystems always had to be established, influenced by a combination of precipitation and temperature. The newly evolved macro-biome of a continental cold steppe (that no longer exists) must have had characteristic macro-ecotones against the (glacially reduced) boreal forests and against the continental meadow steppes of temperate latitudes.

Although not existing under the recent climatic conditions, these macro-ecotones can be modelled based on the zonality of the cold-continental conditions of southern Siberia, northern Mongolia or even Yakutia. Here, many floral and faunal elements can be observed together on species-rich meadow steppes of these regions, species assemblages, which are partitioned to different habitats in eastern Europe like dry steppic grasslands, meadow steppes, damp meadows or even salt meadows. The large number of macro-ecotones with their specific species assemblages is the background for the phenomenon of the evolution of so many species specific biogeographies, but hereby also for the paradigmatic patterns, i.e. the regular existence of micro-refugia. However, this is only a special case of the law of uniformity because even today such micro-refugia with peculiar mixtures of faunal and floral elements exist under analogous climatic conditions.

## Competing interests

The authors declare that they have no competing interests.

## Author’s contributions

Both authors wrote this article in equal parts. Both authors read and approved the final manuscript.

## Author’s information

Thomas Schmitt is Professor of Molecular Biogeography at Trier University. His main scientific interests are biogeography (classical and molecular), ecology (classical and molecular), evolutionary biology, conservation biology as well as the taxonomy of butterflies. He also has a special interest in the high mountain systems of the western Palearctic.

Zoltán Varga is Professor emeritus of Zoology at the University of Debrecen. His scientific focus is on biogeography, evolutionary biology, conservation biology and ecology as well as the taxonomy of butterflies and noctuid moths. He is one of the leading experts for the Carpathian Basin, the Balkan Peninsula and the arid zones of Central Asia.
